# Does the Low-Field MRI Appearance of Intraosseous STIR Hyperintensity in Equine Cadaver Limbs Change when Subjected to a Freeze-Thaw Process?

**DOI:** 10.3390/ani11020475

**Published:** 2021-02-11

**Authors:** Georgina C. A. Johnston, Benjamin J. Ahern, Solomon M. Woldeyohannes, Alex C. Young

**Affiliations:** School of Veterinary Science, The University of Queensland, Brisbane 4343, Australia; georgina.johnston@uq.edu.au (G.C.A.J.); b.ahern@uq.edu.au (B.J.A.); s.woldeyohannes@uq.edu.au (S.M.W.)

**Keywords:** horse, cadaver limb, magnetic resonance imaging, STIR hyperintensity

## Abstract

**Simple Summary:**

Research into the advanced imaging appearance of Thoroughbred racehorse fetlocks is receiving increased attention in an effort to better understand and potentially reduce the occurrence of catastrophic fracture in these horses. Studies in this area commonly use cadaver equine limbs from racehorses and a freeze-thaw process prior to imaging. The low-field magnetic resonance imaging (MRI) appearance of the bones of the fetlock joint is of particular interest in the diagnosis of impending fracture in this location. However, little is known about the effect of the freeze-thaw process on the appearance of certain signal changes (“STIR hyperintensity”) seen commonly in racehorses. This study compares the low-field MRI appearance of short tau inversion recovery (STIR) hyperintensity in the bones of cadaver fetlocks from Thoroughbreds in race training, before and after a freeze-thaw protocol. Fifteen cadaver fetlocks with abnormal bone signal were included in the study. Blinded and unblinded statistical comparisons were made. No overall clinical or statistical difference was detected in intensity and distribution of the STIR signal before and after freeze-thaw. This indicates that the MRI appearance of STIR hyperintensity in freeze-thawed cadaver fetlocks can be considered representative of the appearance of pathology in the recently euthanized horse. This is important information to further advance research in the area of Thoroughbred racing fatality reduction.

**Abstract:**

Equine advanced imaging research involving racehorse fetlock pathology commonly uses cadaver limbs and a freeze-thaw process. The presence of short tau inversion recovery (STIR) signal intensity in the distal third metacarpal/metatarsal bone is of particular interest and may be clinically relevant in the diagnosis of horses at risk of fracture. However, little is known about the effect of the freeze-thaw process on the MRI appearance of STIR hyperintensity in these bones. This study compares the low-field MRI appearance of the distal third metacarpal/metatarsal bone from cadaver limbs of Thoroughbreds in race training before and after a freeze-thaw protocol. Blinded and unblinded comparisons were made using objective SNR values and subjective grading. Fifteen cadaver limbs with STIR hyperintensity in the distal third metacarpal/metatarsal bone were included. No overall clinical or statistical significance was detected in STIR signal intensity and distribution after freeze-thaw. Three limbs from one horse had individual changes in STIR hyperintensity that were hypothesized to be attributable to ante-mortem haemodynamic abnormalities caused by anaesthesia. These results indicate that the distribution and intensity of STIR hyperintensity in freeze-thawed cadaver fetlocks can be considered representative of the appearance of pathology in the recently euthanized horse. However, care should be taken with horse selection and handling of the cadaver limbs to ensure reliable appearance of STIR signal after freeze-thaw.

## 1. Introduction

Research into the advanced imaging appearance of racehorse limbs is essential to better understand the pathophysiology and aetiology of bone fatigue and stress-related injury and so reduce the incidence of catastrophic fracture and associated racing fatality. The fetlock region is an anatomical area of interest as it is the most common location of musculoskeletal injury and breakdown in racehorses. Many advanced imaging fetlock studies use cadaver distal limbs from Thoroughbred racehorses and implement a freeze-thaw process prior to imaging the cadaver limbs [1,2,3]. The practice of freezing and thawing equine cadaveric limbs allows storage and banking of limbs and increased efficiency of the imaging process.

Low-field magnetic resonance imaging (MRI) is one of the advanced imaging modalities being investigated to aid in diagnosis of pathology in the distal limbs of racehorses. It is a relatively widely available, non-invasive modality that can be performed in sedated standing horses, especially in cases with suspected fetlock pathology. A specific MRI sequence, short tau inversion recovery (STIR), suppresses fat signal in tissue, allowing other signal such as fluid to be visualized, and is a common sequence included in a standard fetlock MRI examination. The presence of STIR signal hyperintensity in the distal third metacarpal/metatarsal bone is of particular interest and may be clinically relevant for the diagnosis of horses with stress fracture and for prevention of subsequent catastrophic bone failure [4].

Despite the frequency of equine imaging studies using a freeze-thaw protocol, much is unknown about the effects of the freeze-thaw process on the MRI appearance of STIR hyperintensity in bone in the equine fetlock region. Whilst it has been shown that temperature and handling, among other freeze-thaw process variables, can affect the MRI appearance of equine cadaver limbs [5,6,7,8], these studies mostly focused on the appearance of T1-weighted (T1W) sequences, not STIR sequences. Many of the papers using equine cadaver limbs before and after a standardised freeze-thaw protocol found no differences in MRI appearance for T1W, T2-weighted or T2*-weighted sequences [1,2,3,4,9,10].

Previous research has found that the process of freezing and thawing can affect bone parameters and material properties. One study found a small but significant reduction (12%) in bovine tendon T2*-weighted signal intensity after the first freeze-thaw cycle but not after successive cycles [11]. A spectroscopy study on bone mineral properties in mice found changes in amide levels and crystallinity values after two freeze-thaw cycles [12]. It is possible that tissue changes within bone after a freeze-thaw process may affect the imaging appearance of bone in certain MRI sequences.

There remains a paucity of data regarding the reliability of MRI interpretation after freeze-thawing cadaveric limbs with regards to the appearance of STIR signal in bone. Although STIR hyperintensity was originally referred to as “bone oedema”, subsequent studies have shown this signal change can represent a number of different osseous changes including haemorrhage, oedema, fibrosis and osteonecrosis [13,14]. The transient nature of some of these differentials suggests that STIR signal intensity in the bone of cadaver limbs may be more sensitive and prone to change with the freeze-thaw process than signal intensity in other MRI sequences.

This study aims to investigate the effect of a freeze-thaw protocol on the low-field MRI appearance of STIR hyperintensity in the distal equine metacarpus/metatarsus. We hypothesize that freezing and thawing of a cadaver equine fetlock does not change the distribution or the intensity of STIR hyperintensity in the distal aspect of the third metacarpal/metatarsal bone.

## 2. Materials and Methods

Distal limbs from racing Thoroughbreds in work, euthanized in South-East Queensland, were collected. Limbs were included in the study if they (1) were available for MRI examination within 12 h of death and (2) showed evidence of STIR hyperintensity in the distal third metacarpal/metatarsal bone. Distal limbs with a catastrophic fracture in the fetlock region were excluded. Horse data pertaining to age, signalment and date euthanized was collected. This research received ethics approval from the University of Queensland Animal Ethics Committee (SVS/ANRFA/041/18) and informed consent for collection of the cadaver limbs.

Limbs were secured in a weight bearing position within a low field 0.27T magnet (Hallmarq), and a standard fetlock coil was placed at the level of the fetlock. T1W gradient recalled echo (GRE) and STIR fast spin echo (FSE) sequences of the distal metacarpus/metatarsus were acquired in transverse, frontal and sagittal planes, aligned parallel and perpendicular to the long axis of the third metacarpal/metatarsal bone (Table A1). The inversion time (TI) was adjusted to optimize fat suppression prior to acquisition of the STIR sequences in each limb. Directly following imaging, the limbs were wrapped in plastic and placed in a freezer to be stored at −20 °C for 3 weeks minimum. The limbs were then thawed within the MRI room at approximately 20 °C for 12 h prior to repeat examination. The same STIR FSE and T1W GRE MRI sequences were acquired directly post-thawing. The internal temperature of the third metacarpal/metatarsal bone was recorded before each MRI scan (fresh and thawed) by inserting an intra-medullary temperature gauge from the proximal cut end of the bone to the distal extent of the diaphysis, proximal to the MR imaging field. The temperature gauge was removed prior to MR image acquisition.

**Figure 1:**
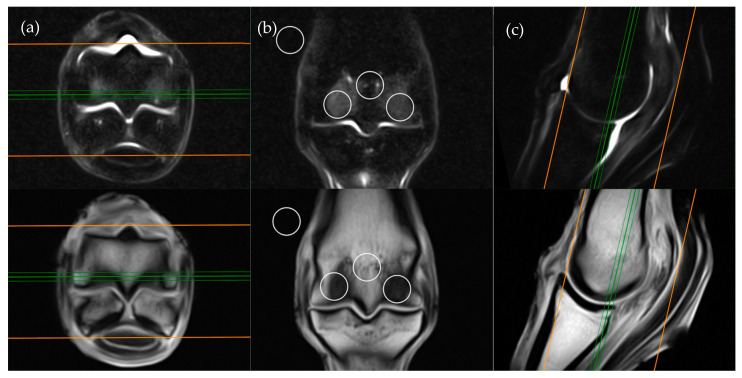
Short tau inversion recovery (STIR) fast spin echo (FSE) (top row) and T1-weighted (T1W) gradient recalled echo (GRE) (bottom row) MRI images in (**a**) transverse; (**b**) frontal and (**c**) sagittal planes; demonstrating the location of ROI placements in a frontal plane. The green lines on the transverse and sagittal images show the standardized orientation and location of the frontal slice for regions of interest (ROI) placement. The orange lines show the extent and orientation of all the frontal slices acquired in the MRI study.

**Figure 2:**
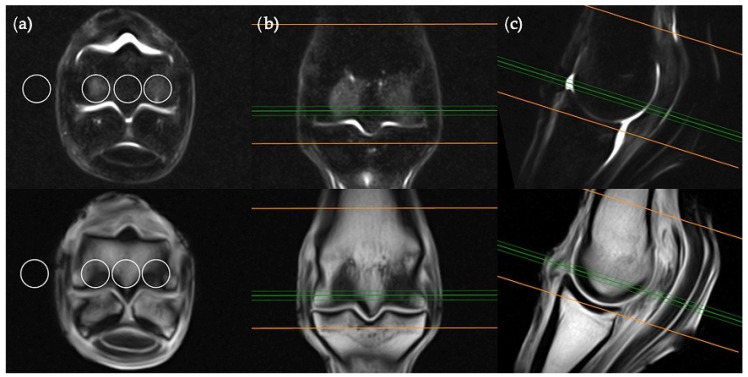
STIR FSE (top row) and T1W GRE (bottom row) MRI images in (**a**) transverse; (**b**) frontal and (**c**) sagittal planes; demonstrating the location of ROI placements in a transverse plane. The green lines on the frontal and sagittal images show the standardized orientation and location of the transverse slice for ROI placement. The orange lines show the extent and orientation of all the transverse slices acquired in the MRI study.

**Figure 3:**
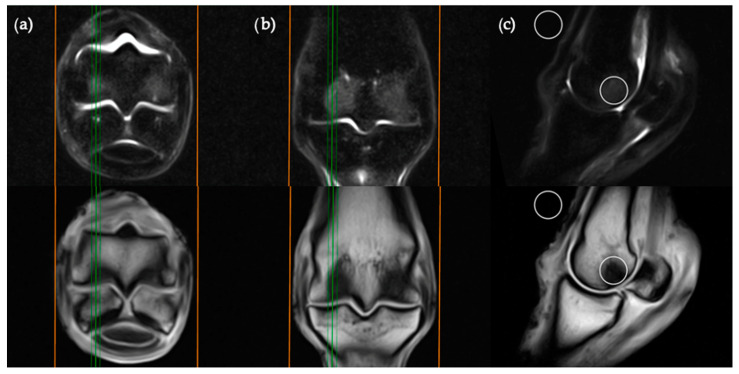
STIR FSE (top row) and T1W GRE (bottom row) MRI images in (**a**) transverse; (**b**) frontal and (**c**) sagittal planes; demonstrating the location of condylar ROI placement in a parasagittal plane. The green lines on the transverse and frontal images show the standardized orientation and location of the medial or lateral condylar parasagittal slices for ROI placement. The orange lines show the extent and orientation of all the sagittal slices acquired in the MRI study.

**Figure 4:**
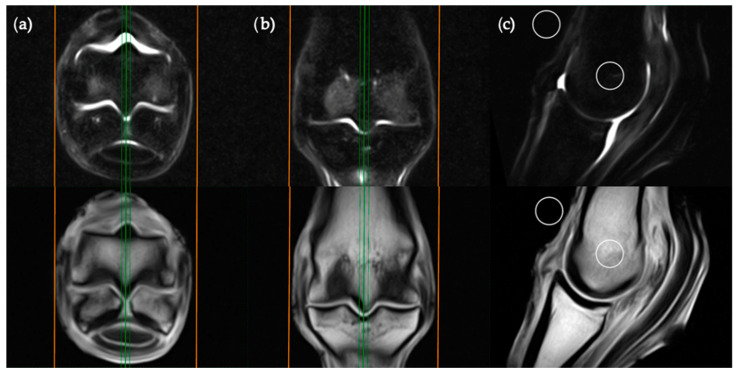
STIR FSE (top row) and T1W GRE (bottom row) MRI images in (**a**) transverse; (**b**) frontal and (**c**) sagittal planes; demonstrating the location of axial ROI placement in a sagittal plane. The green lines on the transverse and frontal images show the standardized orientation and location of the sagittal slice for ROI placement. The orange lines show the extent and orientation of all the sagittal slices acquired in the MRI study.

Images were viewed by a veterinary radiologist experienced with the interpretation of low-field equine fetlock MRI examinations (AY). MRI studies were viewed in “digital imaging and communications in medicine” (DICOM) format on an interactive DICOM viewer workstation (Osirix MD). Standardised regions of interest (ROIs) of 2 cm^2^ were imported into each study. The ROIs were positioned in predetermined locations for each plane of the STIR sequences (Figure 1, Figure 2, Figure 3 and Figure 4) then copied across to the corresponding T1W slice. For every intraosseous ROI measured, a background ROI was also placed external to the anatomy in the same image at a consistent, predetermined location. One to three signal to noise ratios (SNRs) were calculated for each image examined in order to evaluate signal changes between images rather than absolute signal value. SNR is the ratio between the amplitude of the MR signal of a tissue and the standard deviation of the amplitude of the background noise. Use of SNR also allowed for image window and level settings to be adjusted subjectively for each study in order to maximize visualization of intraosseous STIR hyperintensity. Each limb was graded poor, satisfactory, good or excellent for quality of fat suppression in the STIR sequences. The experimental protocol was divided into two parts; blinded and unblinded.

### 2.1. Blinded Comparison

The first part of the experiment was performed objectively by one veterinary radiologist (AY) blinded to the identity and fresh/thawed status of the limbs. Blinding was performed by another investigator (GJ) by randomizing the order of the studies and then removing all identifying details. SNR values were compared between fresh and thawed MRI images at standardised, predetermined locations in the distal third metacarpal/metatarsal bone. These predetermined locations were chosen as those likely to have STIR hyperintensity present and included the distal palmar/plantar region of the lateral and medial condyles and axial physeal region. Each MRI study was assessed in a frontal, transverse and sagittal plane with efforts made to standardize the image chosen for ROI measurement.

The frontal plane image slice immediately dorsal to the corticomedullary junction of the palmar/plantar cortex of the third metacarpal/metatarsal bone was used to obtain ROI measurements at the distal palmar/plantar aspect of the lateral and medial condyles as well as the axial physis (Figure 1). Condylar ROIs were placed at the edge of the subchondral bone plate within the centre of the lateral and medial condyles. The axial physeal ROI was placed on sagittal midline at the level of the physeal vasculature. The background ROI was placed at the lateral or medial surface of the limb proximal to the epicondyle of the third metacarpal/metatarsal bone.

The transverse image located two slices distal to the level of the physeal vasculature was chosen for the transverse plane measurements (Figure 2). Three ROIs were placed; centrally within the distal palmar/plantar margin of the lateral and medial condyles and immediately between these ROIs at the palmar/plantar aspect of the sagittal ridge. The background ROI was placed at the lateral or medial surface of the limb, at the palmar/plantar half of the condyles and in alignment with the three intraosseous ROIs.

The sagittal plane measurements required three different images for ROI placement; parasagittal slices through the centre of the lateral and medial condyles of the third metacarpal/metatarsal bone (Figure 3) as well as a third image on sagittal midline (Figure 4). In the absence of an image perfectly centred of any of these anatomical locations then the adjacent image containing the greatest amount of STIR hyperintensity was chosen. This was commonly required for the sagittal image ROI. Condylar ROIs were placed at the edge of the subchondral bone plate within the centre of the palmar/plantar half of the lateral and medial condyles. The axial physeal ROI was placed within the axial portion of the bone at the level of the physeal vasculature. The background ROI was placed at the dorsal surface of the limb, proximal to the sagittal ridge of the third metacarpal/metatarsal bone.

The ROI measurements on the fresh limbs were performed three times by the same blinded veterinary radiologist (AY) across both STIR FSE and T1W GRE sequences to assess intra-observer reliability. Repeated grading of each limb was performed with an interval of at least one week between image interpretations.

### 2.2. Unblinded Comparison

**Figure 5:**
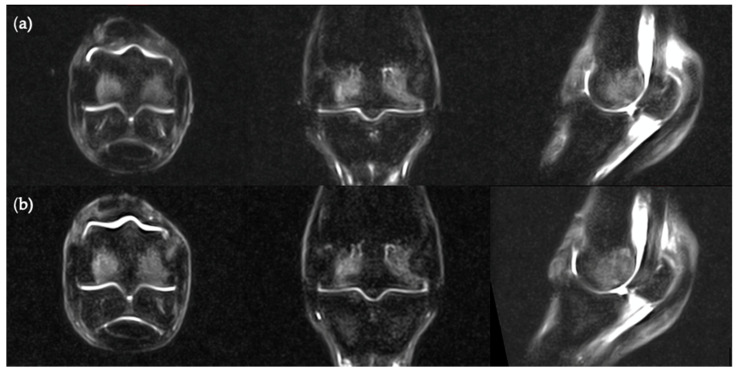
STIR FSE MR images in transverse, frontal and sagittal planes of the same cadaver limb (**a**) after and (**b**) before a freeze-thaw process.

The second part of the experiment was designed to compare and assess the appearance of STIR signal in fresh and thawed limbs in a clinical setting. The same veterinary radiologist (AY) objectively measured SNR and subjectively graded the same limbs using STIR FSE images from co-registered fresh and thawed limbs examined side by side (Figure 5). Window and level settings were not standardised but adjusted to maximize visualization of the STIR hyperintensity. Fresh STIR sequence images were reviewed in frontal, transverse and sagittal planes. The location of maximum STIR hyperintensity within any one imaging plane was chosen for ROI placement (three per limb, one in each plane). An ROI was subsequently placed at the location of minimum STIR hyperintensity on the same image (three per limb, one in each plane). The background ROI was placed at the same standardised location per image plane as that described for the blinded comparison; on the lateral or medial surface of the limb, proximal to the epicondyle of the third metacarpal bone for the frontal plane images, on the lateral or medial surface of the limb, at the level of the palmar half of the condyles for the transverse plane images and on the dorsal surface of the limb, proximal to the sagittal ridge for sagittal images.

The location of each fresh limb STIR FSE ROI was copied across to the corresponding thawed image and manually adjusted to match the location on the fresh image as closely as possible. Some images could not be matched exactly due to differences in slice location between the fresh and thawed limbs. SNR was calculated for each maximum and minimum STIR location. Minimum and maximum locations of STIR hyperintensity were used in order to ensure the study assessed many different intensities of STIR signal. The minimum and maximum STIR hyperintensity was subjectively graded as 0–3 for each plane in both fresh and thawed images (0 = none, 1 = mild, 2 = moderate and 3 = severe). The overall similarity of the fresh and thawed images was subjectively assessed and graded for similarity of distribution (0 = no similarity, 1 = poor, 2 = moderate and 3 = good) and signal intensity (0 = no similarity, 1 = poor, 2 = moderate and 3 = good) of STIR hyperintensity.

### 2.3. Statistical Analysis

Sample estimates were based on preliminary SNR data for mild, moderate and severe STIR hyperintensity. Sample size estimates were conducted using a custom commercial software package designed for power analysis and sample size (PASS, Available online: www.ncss.com, accessed on 15 August 2020). Input assumptions included alpha (0.05), and beta (0.2; equivalent to power of 80%). These input parameter levels are standard for bioequivalence analyses. Summary statistics for mean SNR values and standard deviation were derived for mild, moderate and severe STIR hyperintensity and were used to produce estimates of a coefficient of variation (CoV). Running sample size estimates in PASS for different input CoV values (holding other inputs constant) produced required sample sizes ranging from four to fifteen limbs. These findings suggested that the proposed sample size of n = 15 was likely to detect a change in STIR grade and produce results that were statistically meaningful. Descriptive statistics were used to summarize SNR values for the blinded and unblinded data. An investigator (GJ) not blinded to the identity of the studies collated all the blinded data for statistical comparison between fresh and thawed measurements on the same limb. A Shapiro–Wilks test for normality was performed. For normally distributed data, independent t-tests were performed to assess statistical difference between fresh and thawed data. For comparison of non-normally distributed fresh and thawed data, Mann–Whitney U tests (Wilcoxon rank sum test) were conducted to assess statistical difference. Statistically significant difference was set at 95% (*p* < 0.05).

Based on the outcome of the normality tests, parametric and non-parametric bootstrap methods were used to get intraclass correlation coefficient (ICC) as a measure of intra-observer reliability for the blinded fresh data [15]. A 95% confidence interval was used for the ICC estimate and values less than 0.5, between 0.5 and 0.75, between 0.75 and 0.9 and greater than 0.90 are taken as indicative of poor, moderate, good and excellent reliability, respectively [16].

## 3. Results

Fifteen cadaver distal limbs from five different horses met the inclusion criteria for this study. Of the fifteen limbs, nine were forelimbs and six were hindlimbs. The horses were all Thoroughbred racehorses in active training and aged 3 to 4 years old (mean 3.4 years). Three were geldings and two were fillies. Causes of mortality were sudden death while racing (3/5) or euthanasia due to catastrophic fracture (2/5). Of the horses that were euthanized, one horse had catastrophic fracture in recovery immediately following general anaesthesia for an elective arthroscopy and the other fractured while racing.

The limbs were frozen for 24 to 205 days (mean 75 days). The mean internal temperature of the limbs was 19.7 °C (range 19.5–20.3) in the fresh limbs and 19.9 °C (range 19.8–20.1) in the thawed limbs. The quality of fat suppression on STIR images was subjectively graded good to excellent in all limbs, both fresh and thawed.

Blinded and unblinded data were collected from MRI images of all fifteen cadaver distal third metacarpal/metatarsal bones in the sagittal, transverse and frontal planes both before and after the freeze-thaw process (Tables S1–S3). There was no statistically significant difference in SNR or grade between fresh and thawed limbs for STIR or T1W images with blinded or unblinded comparisons (Table 1, Table 2 and Table 3).

### 3.1. Blinded Comparison

No statistically significant differences were found between fresh and thawed SNR values for all assessed sequences, planes and locations (Table 1).

### 3.2. Intra-Observer Reliaility

The intra-observer reliability for blinded fresh SNR values was variable for different sequences, planes and locations (Table 2). The overall intra-observer reliability for the blinded fresh STIR and T1W SNR data is 0.41 (poor) and 0.76 (good), respectively.

### 3.3. Unblinded Comparison

No statistically significant differences were found between fresh and thawed SNR values nor the subjectively applied grades for any STIR sequence plane (Table 3). The maximum STIR grade ranged from 1 to 3 (mean 2.08) and the minimum grade ranged from 0 to 3 (mean 1.19). Common locations of intraosseous STIR hyperintensity included the axial physis, the centralized portion of the distal palmar/plantar condyles, the dorsal sagittal ridge and the distal dorsal diaphysis of the third metacarpal/metatarsal bone.

Subjectively, twelve of the 15 limbs had good overall similarity with the distribution and relative intensities of the STIR hyperintense regions considered almost identical between the fresh and thawed studies. Two limbs of one horse were graded as good for similarity in STIR signal intensity but moderate for similarity in signal distribution between the fresh and thawed studies. In these two limbs, a mild increase in STIR hyperintensity was visible after the freeze-thaw process at the periphery of the previously identified regions of STIR hyperintensity. A third limb of this same horse was graded as moderate for similarity in STIR signal distribution and poor for similarity in STIR signal intensity due to a marked decrease in STIR hyperintensity following the freeze-thaw process.

## 4. Discussion

This study assessed the effects of a freeze-thaw process on the appearance of STIR hyperintensity in the distal third metacarpal/metatarsal bone of cadaver equine fetlocks. There was no statistical difference in low field MRI appearance of STIR hyperintensity between images acquired from fresh cadaver limbs compared to following a freeze-thaw process. Subjectively, similarity in distribution and intensity of STIR hyperintensity before and after the freeze-thaw process was overall high.

There are a number of variables that may affect the appearance of intraosseous STIR signal intensity in cadaver limbs, including the interval between death and image acquisition, the interval between death and freezing as well as the specific conditions of the freezing and thawing process (e.g., rate, wrapping the limbs, duration and temperature) [5,6,7,11,17,18,19,20,21,22]. This study attempted to standardize many of these variables in order to minimize the potential for intraosseous STIR signal intensity alterations resulting from the freeze-thaw process. The cadaver distal limbs in this study were imaged and frozen within 12 h of death to reduce tissue autolysis. The limbs were wrapped and frozen rapidly to −20 °C to minimize tissue dehydration [17,18]. The limbs were thawed at an ambient temperature of 20 °C in the MRI room for 12 h to reduce cellular damage from a slow, cool thawing process [5,19,20]. Limbs were frozen for 24 to 205 days in this study. Although unlikely to have been significant, it is unknown to what extent the differences in duration of freezing contributed to changes in this study. Duration of freezing time from two weeks to two months has been shown to have no effect on protein denaturation in meat and the duration of freezing was deemed not clinically significant in a previous equine cadaver study [5,23].

The collection of blinded randomised data in this study enabled objective comparison of the SNR values between fresh and thawed images for each limb, sequence and imaging plane. There were no statistically significant differences between any of the SNR measurements. However, intra-observer reliability, although considered good for T1W images, was lower than expected for the STIR images. The lower intra-observer reliability for STIR sequences in the blinded portion of the study were attributed mostly to the slight differences encountered in slice location between the fresh and thawed studies with subsequent differences in ROI location and therefore variations from the effect on volume averaging and the appearance of the vessels.

Although positioning was standardised as much as possible for the blinded measurements, mild variations in image location and placement of the ROIs also likely produced some variability in SNR results. This was particularly evident when the ROIs included physeal vasculature or condylar margins. Vascular channels are markedly STIR hyperintense and large variations in SNR values were observed as the ROI was placed slightly proximally or distally in the most axial location possible. In contrast, the axial SNR measurements of the corresponding T1W images were much more consistent. The small areas of hypointensity associated with these same vascular channels on the T1W GRE sequences were presumed to have had a lesser effect on the corresponding SNR measurements. In contrast to this, condylar SNR measurements obtained in the transverse plane were more consistent on STIR images than the corresponding T1W images. This was likely attributable to the volume averaging present in the palmar condyles, with both hypointense subchondral bone and hyperintense medullary bone included in both the lateral and medial distal palmar/plantar condylar ROI. The corresponding STIR images were more uniformly hypointense in the absence of intraosseous pathology resulting in more consistent SNR measurements from fresh to thawed images in the transverse plane.

Repeatability of manual ROI drawing has previously been found to be good, however repeatability for STIR sequences was not separated from the other MRI sequences in that study [5]. In this study, intra-observer reliability was overall good for T1W measurements, but overall poor for STIR measurements. There was variability in intra-observer reliability found between different planes and locations. As with the blinded comparison, the presence of blood vessels and a slight difference in positioning of a STIR ROI relative to a blood vessel were likely to have contributed to measurement differences. Some slices, particularly for the condyles in the sagittal and frontal planes, may have been different between reads. If the slice did not go directly through the centre of the required anatomy, the slice either side with the most STIR hyperintensity was chosen, however this was subjective and could not be standardised across blinded reads. The intra-observer reliability was lowest on axial STIR ROIs and transverse STIR slices. This was likely due to increased risk of volume averaging in transverse planes and the presence of more blood vessels in axial locations.

Unlike the blinded comparison, the unblinded portion of the study allowed direct side-by-side objective and subjective comparison of the fresh and thawed images of each limb. This allowed us to select areas of STIR hyperintensity as we would in the clinical setting rather than have the location of each measurement predetermined as in the blinded portion of the study. The direct comparison of co-registered fresh and thawed STIR sequences enabled more consistent ROI placement both with respect to image selection and specific ROI placement. This likely minimized variations in ROI positioning and subsequently the effect of volume averaging and blood vessel inclusion.

In the unblinded portion of the study, STIR hyperintensity was commonly identified in the axial physis, distal palmar/plantar aspect of the lateral and medial condyles, dorsal sagittal ridge and distal dorsal diaphysis; areas commonly associated with racing related bone injury. A maximum and minimum STIR hyperintensity were subjectively graded for each image plane so that a range of STIR hyperintensities were assessed (mild, moderate or severe) and so that data was not solely related to areas with marked intraosseous STIR hyperintensity. There was no statistically significant difference found in the subjective grade of the fresh images compared to the thawed images regardless of the severity of STIR hyperintensity.

Subjective and objective comparison of fresh and thawed limbs found no statistical difference in the appearance of STIR hyperintensity, and clinically, there was overall a high similarity between the intensity and distribution of the STIR hyperintensity of fresh and thawed limbs. However, in three of the 15 limbs, there were visible changes in STIR hyperintensity following the freeze-thaw process. Two limbs had a subjective increase in signal at the periphery of the area of STIR hyperintensity when the thawed limbs were compared to the fresh. Another limb from the same horse had a marked reduction in STIR signal intensity with an altered distribution of signal following the freeze-thaw process. Considering these three limbs were collected from the same horse, we hypothesize that the changes in STIR hyperintensity may have been attributable to horse-specific conditions. The horse had been under general anaesthesia in lateral recumbency immediately prior to fracture and subsequent euthanasia. It is possible that this affected the haemodynamics in the distal limbs and predisposed the limbs to fluid changes during the freeze-thaw process. This suggests that limbs from horses euthanized closely following or under general anaesthesia should not be included in cadaver limb MRI research.

This study has some intrinsic limitations. All images were obtained from cadaver fetlocks. Although these results decrease the need to image only “fresh” cadaver limbs when studying the appearance of intraosseous STIR hyperintensity in the fetlock region, we cannot assume this appearance is perfectly representative of the antemortem specimen. Transient causes of intraosseous STIR hyperintensity such as oedema, haemorrhage and inflammatory infiltrate may change rapidly post-euthanasia and could have altered the appearance of STIR hyperintensity in these limbs. However, the locations and distribution of STIR hyperintensity identified in this population were consistent with those seen commonly in our clinical racehorse population.

Previous freeze-thaw studies have used high-field MRI, whereas this study used a 0.27 T low-field magnet. This may have reduced our ability to detect mild changes between fresh and thawed limbs. However, standing low-field MRI is the most commonly used MRI modality for racing Thoroughbreds, and so it is clinically relevant for research with cadaver racehorse fetlocks.

The images of some sequences could not be perfectly co-registered for slice and angle between fresh and thawed MRI acquisitions due to slight differences in limb and image positioning at the time of acquisition. This likely contributed to reduce the similarity between fresh and thawed SNR data and appearance of STIR hyperintensity. There was a small variability in quality of fat suppression between studies. Fat suppression was graded as good to excellent in all image series, however small differences in fat suppression between fresh and thawed limbs may also have affected the appearance of STIR hyperintensity and the SNR values.

## 5. Conclusions

No overall clinical or statistically significant alterations in the low field MRI appearance of STIR hyperintensity were identified in the distal third metacarpal/metatarsal bone of cadaver equine fetlocks following a freeze-thaw protocol. These results indicate that the distribution and intensity of STIR hyperintensity in thawed cadaver fetlocks can be considered representative of the appearance of pathology in the recently euthanized horse. However, it is recommended that cadaver limbs from recently anaesthetized horses are not used in equine MRI research with freeze-thaw protocols. Given the ongoing interest in STIR hyperintensity in equine fetlocks and the use of cadaver limbs in research, further study is required into the effects of handling and environmental conditions on the MRI appearance following freeze-thaw. This information is important to advance further cadaver research into Thoroughbred racing fatality reduction.

## Figures and Tables

**Table 1:** Test of statistical difference between fresh and thawed blinded signal to noise ratio (SNR) values.

Location of Bone ROI	*p*-Value
Sagittal STIR lateral condyle	0.92
Sagittal T1W lateral condyle	0.85
Sagittal STIR medial condyle	1.00
Sagittal T1W medial condyle	0.52
Sagittal STIR axial physis	0.58
Sagittal T1W axial physis	0.61
Frontal STIR lateral condyle	0.65
Frontal T1W lateral condyle	0.51
Frontal STIR medial condyle	0.90
Frontal T1W medial condyle	0.66
Frontal STIR axial physis	0.28
Frontal T1W axial physis	0.71
Transverse STIR lateral condyle	0.79
Transverse T1W lateral condyle	0.38
Transverse STIR medial condyle	0.86
Transverse T1W medial condyle	0.55
Transverse STIR axial physis	0.50
Transverse T1W axial physis	0.72

**Table 2:** Intraclass correlation coefficient (ICC) as intra-observer reliability measure for blinded fresh SNR data.

Location of Bone ROI	ICC for T1W Sequences	ICC for STIR Sequences
Sagittal lateral condyle	0.95	0.51
Sagittal medial condyle	0.64	0.47
Sagittal axial physis	0.76	0.42
Frontal lateral condyle	0.60	0.61
Frontal medial condyle	0.93	0.48
Frontal axial physis	0.78	0.23
Transverse lateral condyle	0.60	0.45
Transverse medial condyle	0.79	0.40
Transverse axial physis	0.78	0.11

**Table 3:** Test of statistical difference between unblinded fresh and thawed STIR hyperintensity grades and SNR values for sagittal, frontal and transverse STIR sequence planes.

Location	*p*-Value for Grade	*p*-Value for SNR
Sagittal-maximum STIR hyperintensity	0.549	0.884
Sagittal-minimum STIR hyperintensity	1.000	0.717
Frontal-maximum STIR hyperintensity	1.000	0.406
Frontal-minimum STIR hyperintensity	0.438	0.340
Transverse-maximum STIR hyperintensity	0.757	0.917
Transverse-minimum STIR hyperintensity	1.000	0.479

## Data Availability

Not applicable.

## Supplementary Materials

The following are available online at https://www.mdpi.com/2076-2615/11/2/475/s1, Table S1: Blinded ROI and SNR measurements from fresh limbs for STIR and T1W sequences in sagittal, frontal and transverse planes. Table S2: Blinded ROI and SNR measurements from thawed limbs for STIR and T1W sequences in sagittal, frontal and transverse planes. Table S3: Unblinded ROI and SNR measurements from fresh and thawed limbs for STIR sequences in sagittal, frontal and transverse planes.

## Appendix A

**Table A1:** MRI sequence parameters.

Imaging Sequence	TR (ms)	TE (ms)	Matrix	Th (mm)	Gap (mm)	Flip (°)	TI	Images in Acquisition
STIR	2862	27	256 × 256	5	0.6	90	65–80	12
T1W	104	8	256 × 256	5	0.6	67	N/A	12
